# Validation of a Graphite Furnace Atomic Absorption Spectrometry (GFAAS) Method for Determining Total Antimony in Organs of Mice

**DOI:** 10.1021/acsomega.5c13656

**Published:** 2026-03-19

**Authors:** Larissa D. Coelho, Mirna M. D. Souza, Maria J. N. Paiva, Clésia C. Nascentes, Mônica C. Oliveira, Marta M. G. Aguiar

**Affiliations:** † Department of Pharmaceutical Products, Faculty of Pharmacy, 28114 Universidade Federal de Minas Gerais, Belo Horizonte 31270-901, Minas Gerais, Brazil; ‡ Department of Clinical and Toxicological Analysis, Faculty of Pharmacy, Universidade Federal de Minas Gerais, Belo Horizonte 31270-901, Minas Gerais, Brazil; § Department of Chemistry, Instituto de Ciências Exatas, Universidade Federal de Minas Gerais, Belo Horizonte 31270-901, Minas Gerais, Brazil

## Abstract

For more than 70 years, antimony (Sb)-based drugs have been the mainstay for leishmaniasis treatment, despite their high cardiac, hepatic, and renal toxicity. In this context, the development of alternative Sb-based formulations remains essential, and liposomal drug delivery systems represent a promising strategy to reduce toxicity and improve therapeutic outcomes. This study addresses the evaluation of Sb quantification by validating an analytical method in various biological matrices—heart, liver, spleen, kidneys, and plasma—using graphite furnace atomic absorption spectrometry (GFAAS). Method validation was performed according to the INMETRO DOQ-CGCRE-008 guideline, assessing the matrix effect, linearity, limit of detection (LoD), limit of quantification (LoQ), accuracy, and precision. Significant matrix effects were observed in all of the evaluated tissues, reinforcing the need for matrix-matched calibration curves. Linearity was demonstrated over the ranges 20–100 μg/L (organs) and 20–120 μg/L (plasma), with correlation coefficients (*r*) exceeding 0.99. LoD values ranged from 0.10 to 3.1 mg/kg, while LoQ values were confirmed between 0.3 and 10.4 mg/kg, with recoveries from 93% to 97%. Accuracy assessments yielded recoveries from 90% to 109%, and precision—evaluated through intra- and interday analyses—resulted in relative standard deviation values below 7% and Horwitz ratio values below 1.0. The method proved to be reliable, reproducible, and sensitive, meeting the validation criteria of the adopted guidelines. As pharmacokinetic and biodistribution profiles of Sb have already been established in our previous work, the validated method presented here reinforces the analytical basis required for ongoing and future preclinical evaluations of antimonial formulations.

## Graphical abstract

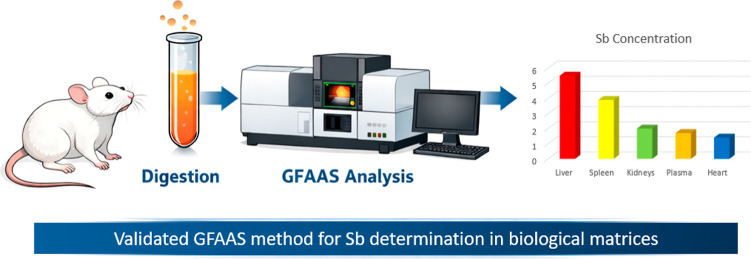

## 1 Introduction

Antimony (Sb) is a semimetal of recognized therapeutic relevance, and its antimonial derivatives remain essential chemotherapeutic agents for the treatment of parasitic diseases such as leishmaniasis.^1,2^ Beyond leishmaniasis, antimony compounds have historically been explored for the treatment of other parasitic and infectious diseases, including schistosomiasis, trypanosomiasis, and certain fungal infections, due to their broad antiparasitic activity. In the context of leishmaniasis, pentavalent antimonials (Sb^5+^) are administered as prodrugs and undergo partial in vivo biotransformation into the trivalent form (Sb^3+^), a process associated with antileishmanial activity but also with increased chemical reactivity and toxicity toward host tissues.^3^ The drawbacks of using antimonial derivatives are related to their narrow therapeutic window, complex biotransformation, and well-documented dose-dependent toxicity, particularly gastrointestinal, hepatic, and cardiac effects associated with trivalent Sb^3+^.^2,4^ These systemic toxic effects are often associated with low and heterogeneous Sb accumulation across organs, making its accurate quantification in complex biological matrices analytically challenging and highly dependent on sensitive, matrix-appropriate methods.^3,5,6^ Preclinical biodistribution and toxicity studies are therefore crucial to better understand Sb disposition and safety profiles.^7^

Visceral leishmaniasis (VL) continues to rely primarily on pentavalent antimonials in many endemic regions.^8–10^ However, their clinical use is limited by painful prolonged parenteral administration, poor adherence, toxicity, and increasing resistance.^8–11^ Although alternative drugs exist, such as amphotericin B and paromomycin, they also require parenteral dosing and present significant adverse effects, reinforcing the need for safer therapeutic approaches. In this context, liposomal formulations have emerged as promising drug delivery systems capable of reducing systemic toxicity and enhancing the therapeutic index. Previous work carried out by our research group demonstrated that liposomes containing tartar emetic (Lip-TE) reduce acute hepatic and cardiac toxicity while maintaining antileishmanial efficacy.^4^ More recently, Lip-TE treatment was shown to improve pharmacokinetic parameters and preserve cardiac function when compared with free TE treatment, further supporting its potential for VL treatment.^12^ The evaluation of such formulations critically depends on accurate and sensitive quantification of Sb in biological matrices, linking therapeutic advances directly to analytical performance.

Accurate quantification of Sb in biological matrices is essential for understanding its absorption, distribution, metabolism, and excretion (ADME) behavior, particularly in preclinical pharmacokinetic and biodistribution studies.^13^ While chromatographic techniques such as LC–MS/MS and HPTLC have been successfully validated for pharmaceutical compounds, their application to total Sb determination may be limited by extensive sample preparation, susceptibility to matrix effects after digestion, and higher operational complexity when applied to inorganic analytes.^14,15^ Graphite furnace atomic absorption spectrometry (GFAAS) remains the preferred technique for total Sb determination due to its high sensitivity, low sample consumption, and suitability for complex matrices.^4,16,17^ The controlled temperature program employed in GFAAS analyses, comprising drying, pyrolysis, and atomization, allows efficient analyte detection even in digested biological samples.^18,19^ Furthermore, instruments equipped with Zeeman background correction improve selectivity in matrices with high organic load.^20^ The applicability of GFAAS for metal and metalloid determination in complex biological tissues has been widely reported, supporting its suitability for preclinical studies of formulations containing Sb.^5,6^ Additionally, the need for reliable analytical methods is reinforced by reports describing tissue-specific toxicity following exposure to metal-based compounds.^21^

International guidelines from AOAC, ICH, and particularly INMETRO, followed in this work, require that method validation include evaluation of linearity, detection and quantification limits, accuracy, precision, and matrix effect to ensure reliable analytical performance.^22–24^ Although Sb quantification methods in biological samples have been previously reported, most validations have been conducted in a limited set of matrices, such as liver, plasma, bone marrow, or skin, from humans or dogs, without extending systematic evaluation to other organs of pharmacokinetic and toxicological relevance, including heart, spleen, and kidneys of mice.^5,6^ Given the growing interest in Sb-based nanoformulations for treatment of VL, and the promising therapeutic profile of Lip-TE, a validated and robust analytical method is required to support forthcoming pharmacokinetic and biodistribution studies.

Thus, the objective of this work was to develop and validate a GFAAS-based analytical method for quantifying total Sb in plasma, heart, liver, spleen, and kidneys of BALB/c mice, and to report its first application to this multiorgan biological system, enabling its application in future studies using treatments with free and liposomal tartar emetic formulations.

## 2 Results

### 2.1 Evaluation of Matrix Effect

The matrix effect was the first parameter evaluated as it directly influences the reliability of quantification and the selection of the calibration strategy. When present, this effect can alter the analytical signal, requiring calibration curves prepared in the same biological matrix (matrix-matched) to ensure accuracy.^22^ To assess this, calibration curves were constructed for each organ (heart, liver, spleen, kidneys) and plasma in both matrix-containing and matrix-free (solvent) media. Each curve was based on five concentration levels (six for plasma) with triplicate measurements. The residuals of the linear regression models were first assessed for normality using the Shapiro–Wilk test. All evaluated residual distributions showed *p*-values >0.05, confirming that the residuals follow a normal distribution, thus validating the use of parametric tests.

Homoscedasticity between curves was evaluated using the Cochran test, with results indicating equal variances across replicates (*p* > 0.05). Subsequently, Student’s *t*-test was applied to compare the mean responses of each fortification level between the solvent and matrix curves. For all evaluated matrices, the *t*-test revealed statistically significant differences (*p* < 0.05), indicating the presence of matrix effects. As a result, matrix-matched calibration curves were adopted for all organs and plasma to ensure reliable quantification. This decision is consistent with international guidelines, including INMETRO^22^ and Eurachem,^25^ which recommend the use of matrix-based calibration in the presence of significant matrix interference.

### 2.2 Determination of the Linearity

Linearity was assessed for each matrix (heart, liver, spleen, kidneys, and plasma) by constructing calibration curves with and without biological matrix interference. Each curve included five concentration levels (six for plasma), analyzed in triplicate, and prepared on different days. The evaluation included both graphical and statistical approaches to ensure the methodological reliability.

Residuals from each regression were first evaluated using the Shapiro–Wilk test, confirming their normal distribution (*p* > 0.05 for all conditions). Cochran’s test was employed to assess the homogeneity of variances across replicate measurements. All results yielded C_cal < C_tab (*p* > 0.05), indicating homoscedasticity and validating the use of ordinary least-squares method. Potential outliers were examined using standardized Jackknife residuals, which did not identify any significant anomalies.^22^

Table 1 presents the regression parameters: slope, intercept, determination coefficient (*R* ^2^), linear correlation coefficient (*r*), and Cochran’s test values. According to the ICH Q2­(R1) guideline (ICH, 2005),^24^ a minimum linear correlation coefficient of *r* ≥ 0.99 is recommended for bioanalytical methods. All matrix types satisfied this criterion with *r* values consistently above 0.995. While the *R* ^2^ reflects the proportion of variance explained by the model, *r* is the primary parameter for assessing linearity acceptance and regulatory compliance.^22^

**Table 1:** Evaluation of the Linearity of the Sb Quantification Method Using GFAAS^a^ ^,^ ^b^ ^,^ ^c^ ^,^ ^d^ ^,^ ^e^

	matrix	linear range (μg/L)	slope (L·μg^–1^)	intercept	*R* ^2^	*r*	Cochran test (calculated values)
heart	solvent	20–100	0.0018	0.0059	0.9989	0.9994	0.4946
	matrix-matched	20–100	0.0015	0.0448	0.9995	0.9997	0.3546
liver	solvent	20–100	0.0062	0.0491	0.9966	0.9982	0.3022
	matrix-matched	20–100	0.0028	0.0626	0.9917	0.9958	0.6103
spleen	solvent	20–100	0.0048	0.0473	0.9991	0.9995	0.6105
	matrix-matched	20–100	0.0043	0.0901	0.9965	0.9982	0.4088
kidneys	solvent	20–100	0.0055	0.0700	0.9979	0.9989	0.4301
	matrix-matched	20–100	0.0051	0.0865	0.9931	0.9965	0.5289
plasma	solvent	20–120	0.0043	0.0309	0.9939	0.9969	0.4282
	matrix-matched	20–120	0.0030	0.0412	0.9913	0.9956	0.3187

a “matrix-matched” refers to curves prepared in digested biological matrices.

b *n* = 3 replicates per concentration level.

c Calibration curves were constructed on separate days for each matrix.

d *r* = linear correlation coefficient; *R* ^2^ = coefficient of determination.

e Cochran test (tabulated values): 0.6160 for organs, 0.5612 for plasma; all C_cal < C_tab (*p* > 0.05).

As detailed in Table 1, matrix effects slightly reduced slope values and increased intercepts, a trend expected due to potential matrix interference. These variations, already discussed in Section 2.1, further support the decision to adopt matrix-matched calibration curves.

Figure 1 illustrates the calibration curves for organs and plasma, respectively. While liver and plasma showed more pronounced matrix effects, the other matrices (heart, spleen, and kidneys) displayed minimal differences between solvent and matrix conditions. Overall, all calibration models demonstrated excellent linearity within the tested ranges (20–100 μg/L for organs and 20–120 μg/L for plasma). These findings are in accordance with Eurachem (2014)^25^ and ICH Q2­(R1)^24^ guidelines.

**Figure 1:**
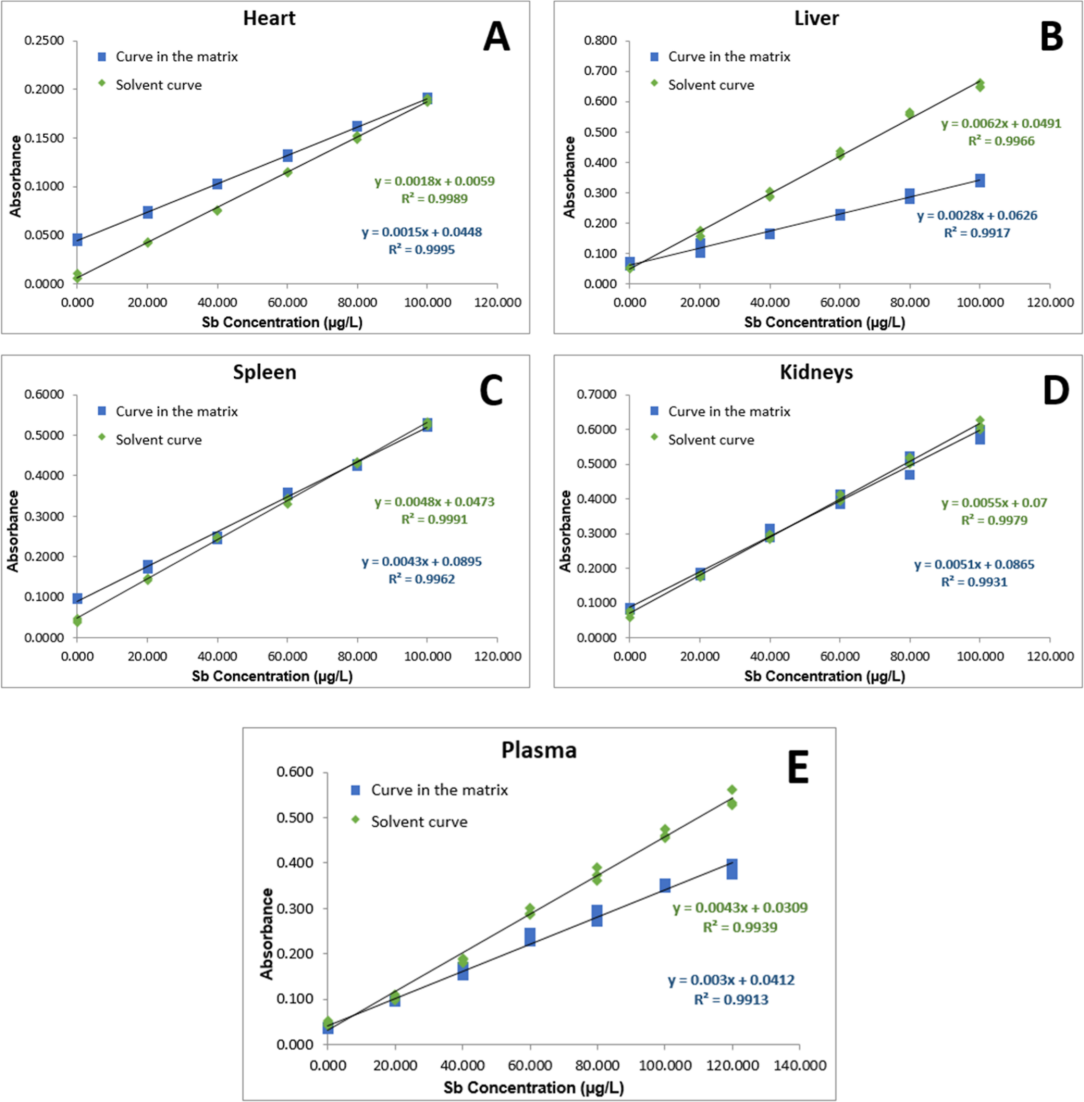
Calibration curves for Sb determination in biological samples. (A) heart, (B) liver, (C) spleen, (D) kidneys, and (E) plasma. Calibration curves were obtained under solvent (green diamonds) and matrix-matched (blue squares) conditions.

### 2.3 Determination of LoD and LoQ

The LoD and limit of quantification (LoQ) were established to determine the analytical method’s sensitivity and its ability to reliably detect and quantify low concentrations of Sb in biological matrices. These parameters do not evaluate the instrument’s efficiency alone, but rather the combined performance of the analytical procedure, encompassing sample preparation, matrix effects, and detection capability.^22^ The LoD is defined as the lowest concentration of the analyte that generates a signal significantly distinguishable from the background noise, whereas the LoQ is the lowest concentration that can be quantified with acceptable precision and accuracy under the specified conditions.^25^

To confirm the LoQ, fortified blank samples were prepared at the respective LoQ concentrations and analyzed in 10 replicates. The recovery rates obtained are shown in Table 2 and demonstrate that the method meets the recovery range recommended by the AOAC (2016),^23^ which stipulates 80–110% for analyte concentrations above 100 ppb. This approach is consistent with regulatory guidelines, including INMETRO for bioanalytical method validation.

**Table 2:** Determination of the LoD and LoQ for Sb Measurement by the GFAAS Method

	standard deviation of blank	LoD (mg/kg)	LoQ (mg/kg)	recovery LoQ (%)
heart	0.3	1.0	3.4	95
liver	1.0	3.1	10.4	97
spleen	0.4	1.3	4.4	96
kidneys	0.3	0.8	2.6	93
plasma^a^	0.03	0.1	0.3	96

a Values for plasma were expressed in mg/L. All LoD and LoQ values were confirmed using *n* = 10 replicates per matrix.

The obtained recovery values (93%–97%) confirm the method’s ability to quantify Sb with accuracy at low concentrations, aligning with findings reported by Azevedo et al. (2013),^5^ who observed 94% recovery at the LoQ level in the skin matrix. These results underscore the method’s sensitivity and reliability across all evaluated biological matrices.

### 2.4 Determination of the Accuracy

In the absence of a certified reference material (CRM) for Sb in the evaluated matrices, the accuracy was assessed through recovery experiments using fortified blank samples. The analyte was externally spiked into each matrix at three concentration levels—low (20 μg/L), medium (60 μg/L), and high (100 μg/L)—with an additional high level of 120 μg/L applied for plasma. Although this approach does not replicate the exact chemical interactions of naturally occurring analyte-matrix binding, it is widely accepted in validation studies and supported by guidelines from INMETRO (2020)^22^ and AOAC (2016).^23^ Each fortified level was analyzed in six replicates (*n* = 6). The average recovery (%) and relative standard deviation (RSD) for each organ and plasma are summarized in Table 3. The recovery values were within the acceptable limits established by AOAC,^23^ which recommends 80–110% recovery for concentrations between 1 mg/kg and 10 mg/kg (with RSD ≤ 11%) and 90–107% recovery for concentrations up to 100 mg/kg (with RSD ≤ 5.3%). These ranges ensure that the method yields accurate and reproducible results across a range of biologically relevant concentrations.

**Table 3:** Determination of Sb Recovery Using Fortified White Matrix and Quantification by the GFAAS Method

matrix	fortified concentration (mg/kg)	concentration recovery (mg/kg)	average recovery (%)	RSD (%)
heart	9.6	9.9	103.8	4.8
	28.1	28.2	100.5	4.3
	47.9	47.6	99.4	1.1
liver	13.0	12.0	90.4	3.9
	43.0	44.0	101.6	3.7
	75.0	76.0	101.9	3.7
spleen	7.3	6.9	94.1	4.9
	21.4	21.9	103.2	2.3
	34.8	34.2	97.9	3.7
kidneys	8.4	8.1	96.1	6.8
	21.4	22.5	105.3	5.7
	43.0	42.9	99.3	4.3
plasma^a^	0.8	0.8	94.3	7.1
	2.4	2.6	109.9	5.0
	4.8	4.5	93.9	4.9

a Values for plasma are expressed in mg/L. RSD = Relative Standard Deviation; *n* = 6 for all matrices.

The recovery rates obtained (ranging from 90% to 109%) confirm that the method delivers accurate quantification of Sb across all tested matrices. These results are consistent with those reported by Azevedo et al. (2013),^5^ who demonstrated recoveries ranging from 94% to 105% in blood and skin matrices. Such consistency reinforces the reliability and applicability of the method in complex biological samples.

### 2.5 Determination of the Precision

The precision of the method was evaluated by analyzing the repeatability (intraday variation) and intermediate precision (interday variation) across three Sb concentration levels—low (20 μg/L), medium (60 μg/L), and high (100 μg/L)—with an additional level of 120 μg/L for plasma. Each level was tested using six replicates (*n* = 6) on two separate days under identical experimental conditions. For each matrix, the mean concentration, standard deviation (SD), RSD, and HORRAT values (i.e., the ratio of the observed RSD to the predicted RSD from the Horwitz equation) were calculated. The *F*-test was applied to compare the variance between the 2 days, and values were interpreted using the associated *p*-values. All statistical analyses were conducted at a 95% confidence level (*p* 0.05).

The results are summarized in Table 4. For concentrations between 1–10 mg/kg, the AOAC (2016)^23^ recommends an RSD ≤ 11%, and for concentrations up to 100 mg/kg, an RSD ≤ 5.3%. All of the tested matrices complied with these limits. HORRAT values remained below 1.0 in the majority of cases, indicating precision superior to that predicted by the Horwitz equation and confirming excellent repeatability and intermediate precision of the method across the evaluated concentration levels.

**Table 4:** Repeatability and Intermediate Precision of the GFAAS Method for Sb Determination Using Fortified Blank Matrices

		first day (repeatability)			second day (intermediate precision)		
matrix	RSD Horwitz	mean (mg/kg)	RSD (%)	HORRAT	mean (mg/kg)	RSD (%)	HORRAT
heart	11.4	9.9 ± 0.5	4.7	0.4	9.5 ± 0.7	7.1	0.6
	9.7	28.2 ± 1.2	4.3	0.4	28.3 ± 0.7	2.6	0.3
	8.9	47.6 ± 0.5	1.1	0.1	47.8 ± 0.3	0.7	0.1
liver	10.9	11.8 ± 0.5	3.9	0.4	12.9 ± 0.5	3.7	0.3
	9.0	44.2 ± 1.6	3.7	0.4	44.8 ± 2.2	4.9	0.5
	8.3	76.3 ± 2.8	3.7	0.4	74.6 ± 2.6	3.5	0.4
spleen	11.9	6.9 ± 0.3	4.9	0.4	7.2 ± 0.2	2.5	0.2
	10.0	22.0 ± 0.5	2.3	0.2	21.6 ± 1.0	4.6	0.5
	9.4	34.2 ± 1.2	3.7	0.4	33.8 ± 1.2	3.6	0.4
kidneys	11.6	8.1 ± 0.5	6.7	0.6	7.7 ± 0.3	3.7	0.3
	10.0	22.5 ± 1.3	5.7	0.6	22.1 ± 1.5	6.6	0.7
	9.1	42.9 ± 1.8	4.3	0.5	43.8 ± 1.6	3.7	0.4
plasma^a^	16.5	0.7 ± 0.1	7.1	0.4	0.8 ± 0.1	10.9	0.7
	14.0	2.6 ± 0.1	5.0	0.4	2.4 ± 0.1	2.5	0.2
	12.6	4.5 ± 0.2	4.9	0.4	4.9 ± 0.3	5.7	0.4

a Plasma values were expressed in mg/L. *n* = 6 for all conditions. RSD = relative standard deviation; HORRAT = RSD observed/RSD predicted by the Horwitz equation.

All of the evaluated matrices showed acceptable RSD and HORRAT values. No statistically significant difference in variance was observed between days (*p* > 0.05), confirming the precision of the method under repeatability and intermediate conditions. As per AOAC (2016),^23^ the method is considered precise across the tested concentration ranges.

### 2.6 Determination of Samples Following Intraperitoneal Administration

To assess the biodistribution of antimony shortly after IP administration of TE, Sb concentrations were quantified in biological matrices at 15 min postdosing. The analysis employed the validated GFAAS method, and the results are presented in Table 5.

**Table 5:** Sb Concentration in Organs 15 min Post-IP Administration

matrix	Sb(mg/kg)
heart	0
liver	25.9 ± 7.4
spleen	4.6 ± 2.1
kidneys	3.9 ± 1.1
plasma^a^	1.5 ± 0.8

a Values for plasma are expressed in mg/L. Data are expressed as mean ± SD (*n* = 7).

As expected, the liver showed the highest Sb accumulation (25.9 ± 7.4 mg/kg), consistent with its central role in the clearance of antimonials and previous observations of preferential uptake in reticuloendothelial organs.^5^ Notably, plasma levels were elevated at this early time point (1.5 ± 0.8 mg/L), supporting the hypothesis that Sb reaches its peak concentration in circulation within the first 15 min post-IP administration.^6^ The spleen and kidneys exhibited intermediate Sb concentrations, while no detectable levels were observed in heart tissue at this time point. The variability observed among organs and between individual animals is likely associated with rapid and heterogeneous absorption from the peritoneal cavity, differences in local blood flow, and individual physiological variability during the early distribution phase. The absence of Sb in the heart may reflect limited early tissue penetration and potential sensitivity limitations of the method near the detection threshold. The considerable RSDs observed in spleen and plasma measurements likely reflect interindividual variability in the early phases of Sb absorption and tissue distribution.^5,6^

## 3 Discussion

The analytical validation of the GFAAS method demonstrated that the proposed procedure is sensitive, and well suited for the quantification of total antimony in digested biological matrices, in accordance with international validation guidelines (INMETRO, 2020;^22^ ICH Q2­(R1), 2005;^24^ AOAC, 2016 ^23^). The complete evaluation of matrix effect, linearity, LoD/LoQ, accuracy, and precision confirms its applicability for subsequent pharmacokinetic, biodistribution, and toxicological studies involving formulations containing antimonial compounds.

A significant observation in this study was the pronounced matrix effect across all evaluated tissues with liver and plasma generally exhibiting more pronounced interference. This behavior can be attributed to their complex biochemical composition, characterized by high concentrations of proteins, endogenous thiols, lipids, and metal-binding biomolecules, which may interact with antimony species and interfere with the atomization efficiency and background correction during GFAAS analysis. In particular, the liver plays a central role in metal metabolism and detoxification, favoring interactions between antimony- and sulfur- or oxygen-containing ligands, while plasma contains abundant proteins and low-molecular-weight ligands capable of altering the analytical response. In contrast, tissues such as heart and kidneys typically present lower levels of these interfering constituents, resulting in comparatively reduced matrix effects.^3,5^

Statistical comparison between solvent-based and matrix-matched calibration curves using the Student’s *t*-test (*p* < 0.05) demonstrated that biological matrices interfere with the analytical response, thereby requiring calibration curves prepared directly in the digested matrix. This result is in accordance with existing literature, in which strong matrix effects have been described for trace metal quantification in blood, skin, and other biological tissues using GFAAS.^5,6^ Biological matrices contain high concentrations of proteins, lipids, salts, and endogenous metals that can alter the atomization efficiency, induce spectral interferences, or modify analyte behavior during thermal treatment. Thus, the adoption of matrix-matched calibration curves ensures more reliable quantification and minimizes systematic bias in biological samples, as recommended by INMETRO^22^ and Eurachem.^25^

The method exhibited excellent linearity within the evaluated ranges (20–100 μg/L for organs and 20–120 μg/L for plasma), with correlation coefficients consistently above 0.995. These values exceed the minimum acceptance criterion of *r* ≥ 0.99 defined by the ICH Q2­(R1) guideline.^24^ Although some matrices, such as liver and plasma, presented slightly lower *R* ^2^ values compared to other tissues, residual analysis confirmed normality, and Cochran’s test indicated homoscedastic variance distribution, validating the use of unweighted linear regression. This behavior aligns with reports that liver digests, due to their high lipid content and complex composition, may introduce additional variability in trace element analysis,^5,26^ further highlighting the importance of matrix-matched calibration.

The LoD and LoQ values obtained demonstrate that the method is sufficiently sensitive to detect low Sb concentrations in all matrices. LoQ verification through recovery experiments yielded recoveries ranging from 93% to 97%, meeting AOAC performance requirements for analyte concentrations above 100 ppb.^23^ These results compare favorably with other studies using GFAAS for metals in biological samples, such as the work by Azevedo et al. (2013),^5^ who reported LoQ recoveries of approximately 94% in the digested skin matrix. The slightly higher LoQ observed for liver (10.4 mg/kg) likely reflects greater background absorbance and matrix complexity due to the high protein and lipid content, a common challenge in bioanalysis of hepatic tissues.

Accuracy assessment demonstrated mean recoveries ranging from 90% to 109% across all matrices and concentration levels, compliant with AOAC acceptance limits.^23^ This high degree of trueness indicates that the digestion procedure, thermal program, and matrix-matched calibration enable the reliable quantification of Sb in complex samples. These results are consistent with previously published studies using GFAAS for biological materials that report similar recovery ranges for metals such as lead, cadmium, and antimony.^5,16,17^

Precision evaluation also confirmed the method’s reproducibility. Both repeatability and intermediate precision displayed RSD values below 7% and HORRAT values <1.0, which are well within AOAC recommendations.^23^ In addition, the absence of statistically significant differences between interday variances (*p* > 0.05, *F*-test) demonstrates that the method performs consistently on different days, an essential requirement for analytical methods intended for routine use in preclinical studies. Such precision performance is comparable to other validated GFAAS methods employing Zeeman background correction for biological matrices, as described in the literature.^17,18^ Overall, the method meets regulatory standards for validation, demonstrating suitability for the quantification of Sb in the heart, liver, spleen, kidneys, and plasma. Its application may contribute to studies of biodistribution, toxicological, and pharmacokinetic involving antimony-based treatments.^12^ These findings align with international practices in trace element bioanalysis and reinforce the method’s utility in preclinical research.

The preliminary biodistribution results obtained 15 min after intraperitoneal administration provide an early indication of Sb distribution kinetics. The predominance of Sb accumulation in the liver reflects the organ’s central function in metal biotransformation and its high density of reticuloendothelial cells.^27^ Intermediate levels in the spleen and kidneys reflect their known involvement in Sb clearance and distribution. The absence of quantifiable Sb in the heart at this early time point is likely due to limited early penetration of the metal into cardiac tissue combined with the vicinity of the method’s LoD. The observed variability in plasma and spleen at 15 min postdose aligns with previous reports describing rapid Sb absorption and pronounced interindividual variation immediately after parenteral administration.^5,6^ These observations justify the importance of using validated sensitive analytical methods to accurately follow Sb kinetics during the early distribution phase.

Although the validated method demonstrated robustness and applicability, the experimental design could be further optimized by anticipating the use of certified reference materials and exploring a broader range of concentration levels and sampling time points. Future investigations may benefit from integrating Sb speciation strategies and chemometric or machine-learning-assisted approaches to improve pattern recognition, predictive performance, and data interpretation in complex biological matrices, as demonstrated in recent analytical studies.^28,29^

Beyond validation, this method provides a crucial analytical foundation for ongoing and future studies involving drug delivery systems containing tartar emetic. Liposomal delivery systems have been shown to influence pharmacokinetic parameters, improve biodistribution, and reduce toxicity of antimonials.^4,12^ Thus, reliable quantification of Sb in tissues is essential to compare formulation performance and elucidate mechanisms underlying reduced cardiotoxicity and prolonged systemic circulation observed in liposomal TE-treated models.

## 4 Conclusions

The validation results confirm that the proposed GFAAS method is suitable for the quantification of Sb in digested biological matrices including the heart, liver, spleen, kidneys, and plasma. The method demonstrated strong linearity, sensitivity, accuracy, and precision across the evaluated concentration ranges, in accordance with regulatory guidelines from INMETRO,^22^ AOAC,^23^ and ICH Q2­(R1).^24^ Matrix-matched calibration curves proved to be essential for mitigating matrix effects, as confirmed by statistical comparison tests. The limits of detection and quantification were sufficiently low to allow detection of trace Sb concentrations, with LoQ recoveries meeting regulatory expectations. Both recovery and precision results further supported the method’s reliability, even in the absence of certified reference materials. Given its performance, this method can be reliably applied in pharmacokinetic, biodistribution, and toxicological studies involving antimony-based compounds.

## 5 Materials and Methods

### 5.1 Materials, Chemicals, and Reagents

Ultrapure water. 65% (v/v) nitric acid solution (HNO_3_) was purchased from Merck (Darmstadt, Germany). Hydrogen peroxide 30% (v/v) was purchased from Labsynth laboratory products Ltda, (Diadema, Brazil). 1000 mg L^–1^ antimony reference solution and zirconium and Triton X-100 were obtained from Merck (Darmstadt, Germany) and were used for sample dilution and preparation of the analytical curve for determination of antimony in plasma. All reagents used were of analytical grade. To prepare phosphate-buffered saline (PBS), phosphate anhydrous monobasic potassium was used which was purchased from Vetec Quimica Fina Ltda, (Duque de Caxias, Brazil); dibasic sodium phosphate heptahydrate and sodium chloride were obtained from Merck­(Darmstadt, Germany); and ethylenediaminetetraacetic acid (EDTA) was purchased from Ecibra (São Paulo, Brazil).

### 5.2 Animals and Housing Conditions

Female BALB/c mice (7 and 8 weeks old) were used in this study. Animals were housed in cages placed within ventilated racks (Alesco ES2 model, Monte Mor, Brazil) with air insufflation, exhaust, and filtration systems, ensuring appropriate environmental conditions in accordance with ethical and animal welfare requirements. The racks were located in a controlled animal facility room with screened windows, regulated temperature, and a 12 h light–dark cycle. Animals were acclimatized in the experimental animal facility for 7 days prior to the experiments. Standard laboratory chow and drinking water were provided ad libitum throughout the study period. Animals used in this study were approved by the Ethics Committee for Animal Experimentation of the Federal University of Minas Gerais (CEUA/UFMG) under protocols *n* ^o^ 240/2018 and 49/2024.

### 5.3 Sample Preparation

#### 5.3.1 Animal Preparation and Sample Collection

Female BALB/c mice were weighed before starting any procedure. For sample collection, animals were anesthetized with a mixture of ketamine (80 mg/kg) and xylazine (15 mg/kg), and blood was collected in tubes containing EDTA (0.1 M). The blood was then centrifuged (3500 rpm, 10 min), and the obtained plasma was frozen at −20 °C. The glassware used was chemically decontaminated by passing through one bath of ultrapure deionized water and one bath of HNO_3_ 10% (v/v) (24 h each) and subsequent rinsing with ultrapure deionized water.

The overall workflow for sample collection, organ digestion, plasma preparation, calibration, and GFAAS analysis is illustrated in Figure 2.

**Figure 2:**
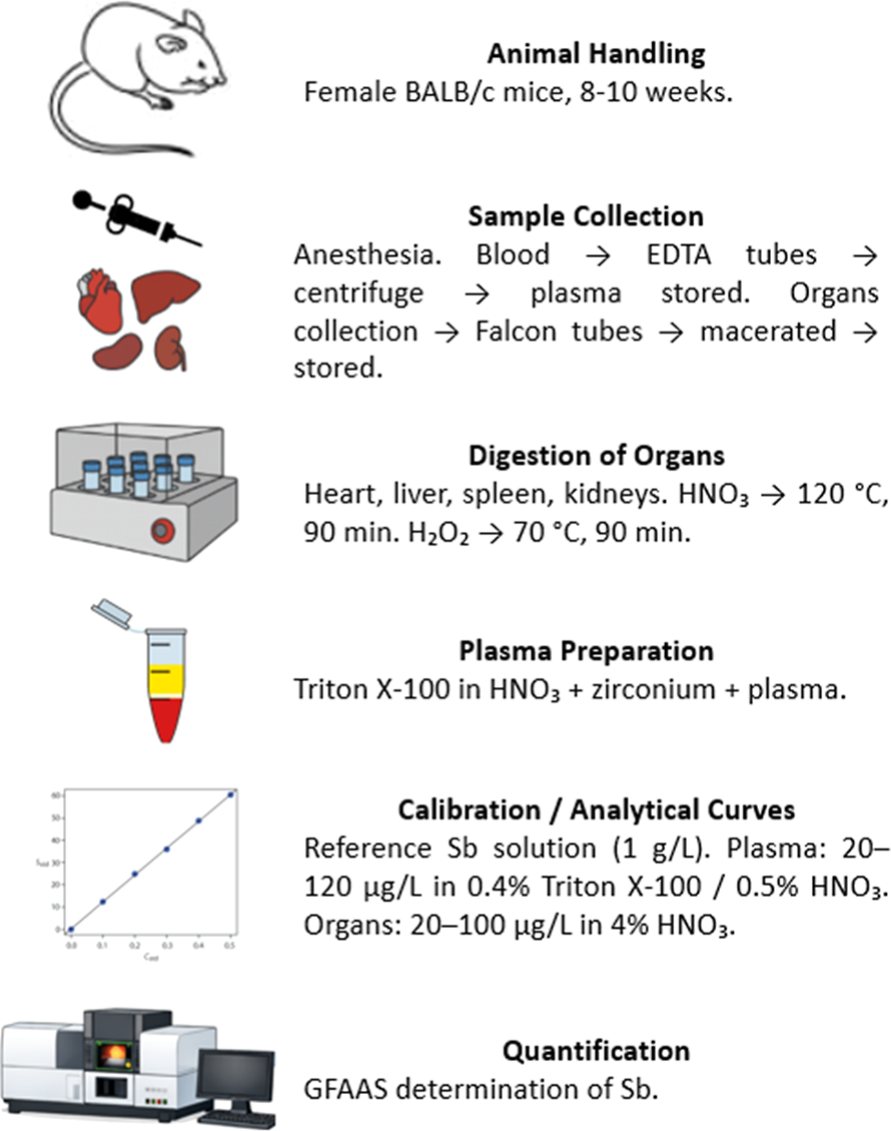
Sample preparation workflow for Sb quantification by GFAAS.

#### 5.3.2 Organ Collection and Homogenization

After euthanasia, organs were transferred to Petri dishes to remove excess blood using PBS and then placed in Falcon tubes containing 2 mL of PBS. After collection, the organs were macerated using an Ultraturrax IKA T25 homogenizer (IKA Labortechnik, Germany) and stored at −20 °C until digestion.

#### 5.3.3 Digestion of Organs

Samples of 1000 mg of heart, 100 mg of liver, 1000 mg of spleen, and 500 mg of kidneys in PBS were digested with 1 mL of 65% nitric acid solution for 1 h and 30 min at 120 °C, followed by the addition of 400 μL of 30% hydrogen peroxide and further digestion for 1 h and 30 min at 70 °C in a digestion block. After digestion, the samples were transferred to volumetric flasks and diluted with ultrapure deionized water to a final volume of 25 mL. The protocol was adapted from the method described by Schettini et al.^13^ This solution was used to prepare the analytical curve and quantify antimony after sample fortification.

#### 5.3.4 Plasma Preparation

Plasma samples were prepared in solutions containing 865 μL (when fortified with the antimony standard) or 965 μL of 0.4% (w/v) Triton X-100 in 0.5% (v/v) HNO_3_, along with 10 μL of zirconium and 25 μL of plasma.

#### 5.3.5 Calibration Solutions and Matrix-Matched Curves

The analytical curves were constructed using a reference antimony solution with a concentration of 1 g/L. Sample fortification was carried out by adding appropriate aliquots of the Sb standard solution directly to blank matrices (previously digested organs or plasma) to obtain a final Sb concentration of 100 μg/L. The fortified blanks were subsequently homogenized thoroughly prior to analysis to ensure uniform analyte distribution and matrix equilibration. For linearity assessment, the working solutions were diluted in 0.5% (v/v) HNO_3_ for plasma comparison and 4% (v/v) HNO_3_ for organ matrices. The standard solution of the analytical curve for Sb in plasma was prepared on the day of the test in a solution of 0.4% (w/v) Triton X-100 in 0.5% (v/v) HNO_3_, with the addition of 10 μL of zirconium and 25 μL of plasma. Calibration points for plasma included 20.0, 40.0, 60.0, 80.0, 100.0, and 120.0 μg/L.

For the quantification of Sb in heart, liver, spleen, and kidneys, the standard solution of the analytical curve was also prepared on the day of the test in digested matrix diluted in 4% (v/v) HNO_3_, consisting of Sb concentrations of 20.0, 40.0, 60.0, 80.0, and 100.0 μg/L. According to Eurachem (2014) guidelines on method validation, calibration curves for linearity assessment should be constructed using a minimum of five concentration levels to ensure sufficient statistical power and reliable regression analysis.^25^ Various studies conducted by different authors demonstrate acceptable results even without using CRM.^21,30,31^

### 5.4 Instrumentation

Absorbance measurements were collected using an atomic absorption spectrometer (Agilent Technologies, Santa Clara, USA, model 240Z AA—200 Series AA), an autosampler (Agilent Technologies, Santa Clara, USA, model PSD 120), and a graphite tube atomizer (Agilent Technologies, Santa Clara, USA, GTA 120) with polarized Zeeman background correction. The system was operated via a microcomputer using SpectrAA software, version 5.2 Pro. Hollow cathode lamps (HCL) for Sb (Agilent Technologies, Mississauga, Canada) were used as a light source. Antimony was determined at 10.0 mA with a spectral bandwidth of 0.2 at 217.6 nm. Argon (99.9992% purity, Air Products, São Paulo, Brazil) was used as a purge gas at a flow rate of 0.3 L/min. Pyrolytic graphite-coated tubes (Agilent Technologies, Mississauga, Canada) were used for analysis. The autosampler injected 17 and 13 μL of the sample into the graphite tube for determination of Sb in organs and plasma, respectively. The signal measurement was based on the peak height, and argon was used as the carrier gas. These methods quantify total antimony without differentiating between its chemical forms. The heating programs for different organs, including predrying, drying, pyrolysis, atomization, and cleaning stages, were optimized based on a previously validated method by our research group^6^ and the manufacturer’s recommendations,^32^ as detailed in Table 6.

**Table 6:** Thermal Program Used for Determination of Sb in Different Organs by GFAAS

	temperature (°C)			argon flow (L/min)
stage	liver time (s)	heart/spleen/kidneys time (s)	plasma time (s)	liver/heart/spleen/kidneys/plasma
predrying	85, 95 (10, 25)	85, 95 (10, 25)	85, 95 (10, 25)	0.3
drying	120 (30)	120 (30, 10)	120 (30)	0.3
pyrolysis	350 (35)	350 (35, 20)	350 (30)	0.3
pyrolysis	750 (10, 5.5)	750 (10, 5.5)	750 (10, 2.5)	0.3
atomization	2000 (0.7, 2)	2000 (0.7, 2)	2000 (0.7, 2)	0
cleaning	200 (3.1)	2000 (3.3)	2000 (2)	0.3

### 5.5 Validation of the Analytical Method

The validation was performed in accordance with the guideline provided by the National Institute of Metrology, Quality and Technology (INMETRO, DOQ-CGCRE-008, 2020),^22^ with support guidelines Eurachem (2014),^25^ and the ICH Q2­(R1) guideline.^23^ The following parameters were evaluated: matrix effect, linearity, LoD and LoQ, accuracy (recovery), and precision (repeatability and intermediate precision) as they are essential to ensure method selectivity in complex biological matrices, proportional analytical response over the working range, adequate sensitivity for low antimony concentrations, trueness of measurements, and reproducibility under repeat and intermediate conditions.^22^ All statistical evaluations were conducted at a 95% confidence level (*p* 0.05).

#### 5.5.1 Matrix Effect

To assess the matrix effect, calibration curves were constructed both in the biological matrices (plasma, digested heart, liver, spleen, and kidneys) and in solvent (nitric acid 0.5% for plasma and 4% for organs). Each calibration level (20, 40, 60, 80, and 100 μg/L; plasma extended to 120 μg/L) was prepared in triplicate. Cochran’s test confirmed homoscedasticity of the data. Student’s *t*-test compared (*p* 0.05) the matrix and solvent curves. The concentration ranges selected for calibration were defined based on antimony levels reported in previous preclinical studies and on the expected concentrations in plasma and organs following intraperitoneal administration at the therapeutic dose used in this study, ensuring relevance for pharmacokinetic and biodistribution analyses.^4–6^

#### 5.5.2 Linearity

The linearity was evaluated by analyzing residuals, the determination coefficient (*R* ^2^), and the linear correlation coefficient (*r*). Normality of residuals was verified using the Shapiro–Wilk test. Cochran’s test evaluated homoscedasticity (*p* 0.05). The presence of outliers was assessed using standardized Jackknife residuals (*p* 0.05). Calibration curves were constructed using five points (six for plasma) in accordance with Eurachem recommendations.^25^

#### 5.5.3 Limits of Detection (LoD) and Quantification (LoQ)

LoD and LoQ were calculated from 10 blank matrix replicates using eqs 1 and 2, respectively.

1 $$LoD=\mathrm{X̅}+t_{\left(9^{,},95\%\right)}.s$$

2 LoQ=X̅+10.s

where *X* is the mean of blank sample values, *s* is the standard deviation, and *t* is Student’s *t*-value for 9 degrees of freedom at 95% confidence. The LoQ was experimentally verified through 10 replicate analyses of the fortified samples. All methods are in line with INMETRO and AOAC (2016) guidelines.^22,23^

#### 5.5.4 Accuracy

Accuracy was determined by spiking samples prior to digestion at three concentration levels (20, 60, and 100 μg/L for organs; up to 120 μg/L for plasma). Six replicates were analyzed per level. All within AOAC-accepted limits for bioanalytical methods.^23^ The recovery experiments were performed following standardized validation procedures using predefined concentration levels; although analyses were not conducted in a blinded manner, this approach is consistent with internationally accepted analytical validation guidelines.^22–25^

#### 5.5.5 Precision (Repeatability and Intermediate Precision)

Precision was assessed at the same three concentration levels as accuracy. Six replicates were analyzed on two different days. RSD, and Horwitz ratio (HORRAT) values were calculated for both repeatability and intermediate precision. The *F*-test was used to compare interday variance.

#### 5.5.6 Statistical Analysis

All statistical analyses related to method validation were performed using GraphPad Prism 8.0 and Microsoft Excel. The Shapiro–Wilk test was used to assess residual normality, and Cochran’s test was used for homoscedasticity. Student’s *t*-test for comparison between matrix-matched and solvent-based calibration curves, *F*-test for evaluation of interday variance, HORRAT calculations, and complementary validation parameters were performed using Microsoft Excel, in accordance with INMETRO guideline recommendations. The presence of outliers was verified by standardized Jackknife residuals. A 95% confidence level was considered, with significance set at the *p*-value (*p*) < 0.05.

### 5.6 Analysis of Samples Following Intraperitoneal Administration

To provide preliminary insights into the biodistribution profile, healthy female BALB/c mice (7 to 8 weeks old) were divided into two experimental groups (*n* = 7 per group). One group received a single intraperitoneal (IP) dose of tartar emetic solution (TE) equivalent to 8 mg/kg of Sb, while the control group was administered only with PBS. 15 min after treatment, the animals were euthanized, and samples of plasma, heart, liver, spleen, and kidneys were collected for Sb quantification. For calibration purposes, organ samples from the control group were fortified with a standard Sb solution to construct matrix-matched calibration curves, a procedure required to compensate for matrix effects and to ensure accurate quantification rather than reflecting insufficient analytical sensitivity.
